# Predictive immune biomarker signatures in the tumor microenvironment of melanoma metastases associated with tumor-infiltrating lymphocyte (TIL) therapy

**DOI:** 10.1186/2051-1426-2-S3-P243

**Published:** 2014-11-06

**Authors:** Jieqing Chen, Caitlin Creasy, Carlos Antonio Torres-Cabala, Suhendan Ekmekcioglu, Sourindra N Maiti, Charuta Kale, Cara Haymaker, Jason Roszik, Roland L Bassett, Jianhua Hu, Zhiqiang Wang, Wencai Ma, R Eric Davis, Chantale Bernatchez, Patrick Hwu, Laszlo Radvanyi

**Affiliations:** 1 M.D. Anderson Cancer Centre (MDACC), University of Texas, Houston TX, USA; 2 Lion Biotechnologies, Woodland Hills, CA, USA; 3 Moffitt Cancer Center, Tampa, FL, USA

## 

Adoptive cell therapy using autologous TIL is a promising immunotherapy for metastatic melanoma. This study was aimed at finding predictive biomarkers in TIL patients, using IHC and gene expression analysis on original tumors used to expand TIL. The purpose was to find factors associated with optimal TIL outgrowth from melanoma tumors and ultimately clinical response to therapy. We found a significant differences in CD8, CD4 and CD3 staining in the tumors between good TIL growers and poor growers by IHC (p < 0.0001, respectively). Interestingly, the CD8 expression in the original tumors also correlated with the percentage of CD8+ T cells in the final TIL product after expansion. Using a new droplet digital PCR assay using TCR Vβ-specific primers called QuanTILfy™, we also found that good TIL growers had a higher TCR Vβ gene signal than samples from poor growers (p = 0.008 Mann-Whitney test) suggesting that this genetic test may be useful in selecting patients for TIL therapy. Gene expression profiling of 595 immunologically-relevant genes found differences in a number of genes in tumors of patients having good TIL growth versus poor TIL growth, such as CD8β and CD3δ, CD45RA, ICOS, PD-1, STAT4. Interestingly, CD8 gene expression was significantly correlated with immunosuppression pathway genes, such as PD-L1, PD-1, FoxP3, and IDO that was confirmed by IHC analysis. Strikingly, IHC analysis of these markers in original tumors, together with CD8, were also found to be predictive of overall survival (OS) after TIL infusion. There were also differences in key genes in tumors of clinical responders and non-responders to TIL treatment. One gene, IRAK1, was particularly interesting as it regulates NFkB signaling and pro-inflammatory cytokine production associated with decreased anti-tumor adaptive immunity. Both gene expression and IHC analysis found an association between higher IRAK1 and decreased OS and relapse-free survival after TIL infusion. In conclusion, our IHC and gene profiling assays on archived tumor samples have uncovered predictive biomarker signatures identifying patients eligible for TIL therapy and who will most likely benefit from TIL therapy, as well as possible new tumor resistance pathways to immunotherapy for melanoma that can be targets of novel combination therapies to enhance TIL therapy.

